# Incidence and evolution of venous thrombosis during the first 3 months after irreversible electroporation of malignant hepatic tumours

**DOI:** 10.1038/s41598-019-56324-y

**Published:** 2019-12-27

**Authors:** W. Bäumler, M. Sebald, I. Einspieler, P. Wiggermann, A. Schicho, J. Schaible, L. Lürken, M. Dollinger, C. Stroszczynski, L. P. Beyer

**Affiliations:** 1 Department of Radiology, University Hospital Regensburg, Regensburg, Germany; 2 Department of Radiology, Municipal Hospital Landshut, Landshut, Germany; 3 Department of Radiology and Nuclear Medicine, Hospital Braunschweig, Braunschweig, Germany

**Keywords:** Hepatic portal vein, Magnetic resonance imaging, Outcomes research, Cancer, Surgical oncology

## Abstract

The incidence and evolution of venous thrombosis adjacent to the ablation zone after percutaneous irreversible electroporation (IRE) were evaluated to identify potential risk factors in patients with hepatic malignancies. 205 venous structures (in 87 patients) within a ≤1.0 cm radius of the ablation zone were assessed after IRE of 112 hepatic lesions (74 primary, 38 secondary hepatic malignancies) by pre-interventional and post-interventional (1–3 days, 6 weeks and 3 months after IRE) contrast-enhanced magnetic resonance imaging. The relationships between venous thrombosis and clinical features were analysed using a binary logistic regression model. In 27 of 87 patients (31%), a total of 67 venous complications were noted during the 3 months follow-up. Thrombosis represented the most frequently observed complication (n = 47; 70.1%), followed by vessel narrowing (n = 20; 29.9%). 5 (10.6%) of 47 thromboses showed spontaneous regression 3 months after IRE. A small vessel diameter (p = 0.011) and post-interventional vessel narrowing (p = 0.006) were independently associated with delayed post-ablative thrombosis. Delayed venous thrombosis frequently occurs after IRE of hepatic malignancies. Pre-existing vessel narrowing and a small vessel diameter represent significant risk factors that require further surveillance and potentially therapeutic intervention.

## Introduction

In recent years, percutaneous thermal ablation of liver tumours has become increasingly established in the clinical routine because of its significant efficacy and safety improvements. It has played a key role in the management of hepatocellular carcinoma and colorectal cancer liver metastases.

Although ablation technology has evolved with the development of high-energy microwave ablation systems, the risk of complications or technical failure after local ablation therapy is still increased for the treatment of hepatic tumours in critical locations such as those adjacent to intrahepatic vessels^1^. First, there is a risk of vessel damage^2^. Second, the “heat sink effect”, which represents the loss of heat owing to the flow of blood in adjacent vessels^3,4^, can affect the size of the ablation zone^5^, potentially leading to residual tumour near the vessel.

Irreversible electroporation (IRE), as a predominantly non-thermal ablative method, represents a viable alternative to thermal ablation; cell death is achieved through repeated application of high-voltage electrical impulses that create irreversible damage to the membranes of tumour cells^6^. Compared with thermal ablation methods such as microwave and radiofrequency ablation, no significant difference in the length of hospital stay, intensive care unit stay and the occurrence of post-ablation syndrome has been observed^7^. Constituting a potent and safe ablative method that protects the architecture of adjacent vascular structures^8,9^, IRE seems to have an advantage over thermal ablation techniques. However, alterations such as thrombosis or narrowing of vessels adjacent to the ablation zone have been reported in a few cases^10,11^. Although the immediate occurrence of post-ablative vessel complications has been investigated by several authors, little is known about the delayed onset of thrombosis during intermediate-term follow-up.

The aim of this study was to evaluate the incidence and evolution of thrombosis in venous structures adjacent to the ablation zone after IRE in patients with hepatic malignancies during the first 3 months after intervention. Furthermore, the relationship between venous complications and clinical features was analysed.

## Methods

### Study design, participant selection and patient characteristics

The retrospective single-centre observational study was approved by the Ethics Committee of the University Regensburg (approval number 18-1027-104). It was performed in accordance with the relevant guidelines and regulations. Written informed consent was obtained from all patients for the acquisition of pre- and postinterventional contrast-enhanced MR images, the ablation procedure and the anonymous use of the data for scientific purposes. MRI images obtained between December 2011 and May 2018 were retrospectively evaluated for complications in venous structures adjacent to the ablation area after percutaneous IRE. Concerning the potential changes in vessels, the authors mainly concentrated on venous thrombosis. Moreover, cases of post-ablative vessel narrowing were also considered to investigate the potential influence on the occurrence of thrombosis. The inclusion criteria of the study were: (I) Primary or secondary liver malignancies treated by percutaneous IRE including patients that had been previously treated with any other kind of therapy (e.g., surgery, systemic chemotherapy). (II) Examination of the entire liver by contrast-enhanced MR images during the portal venous phase before and after IRE, i.e., 1–3 days, 6 weeks and 3 months after the ablation. (III) Venous structures (portal veins [PVs], hepatic veins [HVs], inferior vena cava [IVC], transjugular intrahepatic portosystemic shunt [TIPS] and umbilical vein [UV]) were located within a radius of 1.0 cm of the ablation area at follow-up MR imaging examination after the intervention. The vessel-ablation zone distance was graduated into three categories: encased, i.e., completely surrounded by the ablation zone; abutting, i.e., directly adjacent to the ablation zone, but not encased; and distant, i.e. 0.1–1.0 cm from the ablation zone.

A total of 87 patients (67 men and 20 women) aged 66.2 ± 11.7 (range, 35–90 years) fulfilled the inclusion criteria. IRE ablation was performed in 112 lesions in 91 ablation procedures (Table 1). In 24 patients only one follow-up control (1–3 days after IRE) could be acquired. The mean tumour diameter was 2.1 cm ± 1.0 (range, 0.4–4.4 cm). 74 of 112 lesions (in 57 patients) were primary liver tumours, 38 of 112 lesions (in 30 patients) were secondary liver tumours (Table 2). A single tumour was ablated in 87 sessions, whereas two tumours were ablated in 4 sessions. Of the 87 treated patients, 21 received one or more chemotherapy cycles before ablation (24.1%). In the subgroup of patients with colorectal liver metastases, 17 of the 21 patients received prior chemotherapy (81.0%).

**Table 1:** Baseline patient and disease characteristics.

Characteristics	
Age (y)	
Mean ± SD	66.2 ± 11.7
Range	35–90
Sex, n (%)	
Male	67 (77.0)
Female	20 (23.0)
Tumour diameter (cm)	
Mean ± SD	2.1 ± 1.0
Range	0.4–4.4
Patients with liver cirrhosis, n (%)	51 (58.6)
Tumour localization, n (%)	
Segment I	4 (3.6)
Segment II	13 (11.6)
Segment III	9 (8.0)
Segment IVa	12 (10.7)
Segment IVb	14 (12.5)
Segment V	22 (19.6)
Segment VI	12 (10.7)
Segment VII	3 (2.7)
Segment VIII	23 (20.5)

SD = standard deviation.

**Table 2:** Tumour types of 87 patients treated with irreversible electroporation of malignant liver tumours.

Diagnosis	Number of patients	Number of treated lesions
*Primary liver tumours*		
HCC	50	65
CCC	7	9
*Metastasis of*		
Colorectal tumour	21	26
Mammarian carcinoma	3	4
Others*	6	8
**Total**	**87**	**112**

*Carcinoma of unknown origin, neuroendrocrinic tumour.

The pre- and post-interventional MR images were analysed by two radiologists with 4 years and 7 years of experience in abdominal imaging. Each MR image was examined for possible vascular changes in diameter and patency by consensus reading. During follow-up scans, the evolution of vessel alterations was assessed. The MR protocol included the following sequences: T2 haste, T1 vibe3d in-phase, T1 vibe3d out-of-phase, T1 vibe3d fat suppressed, T1 vibe3d fat suppressed contrast-enhanced early arterial phase, T1 vibe3d fat suppressed contrast-enhanced late arterial phase, T1 vibe3d fat suppressed contrast-enhanced portal venous phase, T2 blade fat suppressed contrast-enhanced, diffusion trace contrast-enhanced and T1 vibe3d fat suppressed delayed phase.

### Irreversible electroporation

All IREs were performed percutaneously under computed tomographic fluoroscopy guidance and full anaesthesia with deep muscle relaxation using the NanoNife system (AngioDynamics, Latham, New York). The operator placed two to six monopolar 18-gauge IRE probes parallel to each other in or around the target tumour. The IRE parameters were as follows: pulses per cycle, 70; pulse length, 90 µs; electric field, 1500 V/cm needle distance. A test pulse of 270 V was delivered before the delivery of the 90 therapeutic pulses to confirm sufficient conductivity.

### Statistical analysis

All collected data are presented as frequency counts and percentages. A binary logistic regression model was used to identify variables for prediction of thrombosis at the mentioned specific points of follow up. Equal correlation of the binary response for individual patients was assumed, implying an exchangeable correlation structure. As effect estimates, maximum-likelihood odds-ratio estimators and 95% confidence intervals are presented. A p-value of ≤0.05 was considered statistically significant. To analyse the correlation between the occurrence of late thrombosis and diameter of the vessel, type of vessel (portal venous system vs. hepatic veins), vessel-ablation zone distance, previous post-ablation narrowing of the vessel and previous chemotherapy, a general estimating equation for logistic regression (GEE) was applied. The model was fitted under the assumption that the probability of a thrombosis of one vein cannot be considered to be statistically independent of the thrombosis of other veins in the same patient. Therefore, the patient was added as a random effect to the model. The GLMM analysis was performed using R 3.5.2 and Zelig. All other statistical analyses were performed with SPSS statistic (IBM SPSS Statistics, version 25).

## Results

**Figure 1:**
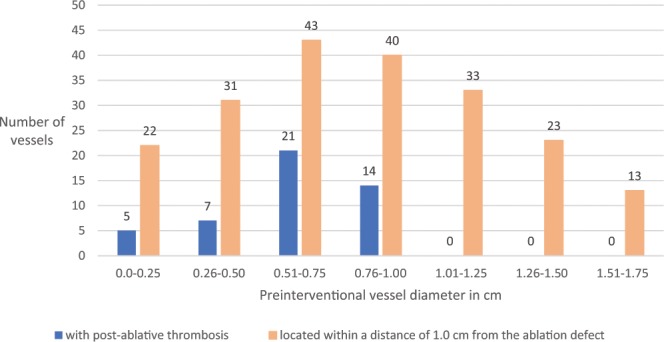
Distribution of post-ablative thrombosis relative to vessel diameter.

In this study, 205 venous structures in 87 patients were located within a distance of 1.0 cm from the ablation defect. 79 (38.5%) were encased by the ablation zone, 101 (49.3%) abutted the ablation defect and 25 (12.2%) were located within a radius of 0.1–1.0 cm of the ablation area. An overview of the details regarding vessel type and complications is presented in Table 3. In total, 67 vascular complications were noted in 27 patients (31%). In 28 of the 205 veins (24 patients) located within a radius of 1.0 cm, only one follow-up control (1–3 days after IRE) was acquired. Of those, 25 (21 patients) did not show any vascular complications, whereas thrombosis was observed in 2 vessels (1.0%; 2 patients) and narrowing was observed in 1 vessel (0.5%; 1 patient). The remaining 177 venous structures (63 patients) were evaluated at each of the defined points of time. The different types and the development of vascular complications are listed in Table 4. Thrombosis (n = 47; 70.1%) was the most frequently observed complication, followed by vessel narrowing (n = 20; 29.9%). Veins with a distance of 0.1 cm or more from the ablation zone were not affected by thrombosis. Furthermore, all post-ablative thromboses were observed in vessels with a maximum pre-interventional diameter of 1.0 cm. An overview of the distribution of thrombosis relative to vessel diameter is provided in Fig. 1.

**Table 3:** Types and numbers of vessels adjacent to the ablation zone and numbers of vessels with vascular complications with regard to their localization.

Vessel	Total number of vessels/number of vessels with vascular complications	Number of vessels adjacent to the ablation zone/number of vessels with vascular complications		
		Encased/Complication	Abutting/Complication	Within a radius of 0.1–1.0 cm/Complication
Main PV	4/2	0/0	2/2	2/0
Left PV or segmental PV branch	45/17	17/9	21/7	7/1
Right PV or segmental PV branch	75/27	31/15	31/9	13/3
Middle HV	23/9	13/7	10/2	0/0
Left HV	19/6	10/5	9/1	0/0
Right HV	27/6	5/2	20/4	2/0
IVC	7/0	1/0	5/0	1/0
TIPS	3/0	2/0	1/0	0/0
UA	2/0	0/0	2/0	0/0
Total	205/67	79/38	101/25	25/4

PV, portal vein; HV, hepatic vein; IVC, inferior vena cava; TIPS, transjugular intrahepatic portosystemic shunt; UA, umbilical vein.

**Table 4:** Numbers and types of vascular complications during the first 3 months after IRE.

	Day 1–3	6 weeks				3 months				Complications (total)
	Complications	Complications				Complications				
	New	Number	New	Resolved	Change	Number	New	Resolved	Change	
Thrombosis	17*	38	23	0	0	40	7	5	0	47
Vessel narrowing	19**	5	1	11	3	1***	0	2****	2	20
										67

IRE, Irreversible Electroporation; *2 thromboses without follow-up 6 weeks and 3 months after IRE; **1 vessel narrowing without follow-up 6 weeks and 3 months after IRE; ***in this case vessel narrowing remained unchanged during the whole follow-up period; ****One of the two cases of vessel narrowing was detected 6 weeks after IRE.

### Delayed thrombosis

23 of all thromboses (48.9%) were detected 6 weeks after IRE, whereas 7 thromboses (14.9%) occurred for the first time between 6 weeks and 3 months after IRE. In 5 of 20 cases (25.0%), vessel narrowing turned into thrombosis 6 weeks (n = 3) or 3 months (n = 2) after IRE (Table 4). A small vessel diameter and post-interventional vessel narrowing were significantly unfavourable prognosticators of delayed thrombosis (p = 0.012 and p = 0.004; Table 5).

**Table 5:** Results of binary logistic regression model predicting delayed thrombosis, i.e., thrombosis occurring 6 weeks to 3 months after IRE.

Predictor	Estimate	Standard error	p-value
(Intercept)	−1.4260	0.5886	0.0154
Vessel narrowing			
No	Reference		
Yes	1.4219	0.5104	0.0053
Vessel type			
Hepatic vein	Reference		
Portal vein	−0.3376	0.4473	0.4503
Vessel-ablation distance			
0.1–1.0 cm	Reference		
Abutting or encased	0.3761	0.4958	0.4481
Diameter (mm)	−1.3386	0.6595	0.0424
Previous chemotherapy			
No	Reference		
Yes	0.2853	0.4489	0.5250

### Evolution of thrombosis

11 (16.4%) complications (vessel narrowing; n = 11) resolved during the first 6 weeks, whereas 7 (10.4%) pathological alterations (thrombosis: n = 5; vessel narrowing: n = 2) showed spontaneous regression 3 months after the intervention (Table 4).

## Discussion

This is the first major study that systematically addresses the incidence of delayed post-IRE venous thrombosis. Vascular changes after IRE appear to occur in 31% of patients, which is more frequently than previously assumed^11^. We also showed that narrowing of blood vessels after IRE progresses to thrombosis in 25.0% of all cases, representing an independent risk factor. Furthermore, the study suggests that a small vessel diameter may increase the risk of delayed thrombosis after IRE.

Although surgical resection is often considered the most effective treatment method for primary or secondary hepatic malignancies because they are associated with the best results with regard to survival, many patients cannot be treated by resection because the tumour is too advanced or because of several comorbidities^12^.

Percutaneous electrochemotherapy as a tumour treatment method, combining the use of electric pulses and cytotoxic drugs^13^, represents a novel and probably very effective alternative for minimally invasive oncologic treatment of solid organs^14^. An animal study performed by Zmuc *et al*.^15^ indicated that electrochemotherapy with bleomycin seemed to be a safe treatment option for hepatic tumours preserving large intrahepatic vessels and bile ducts. However, there is still a lack of data on the effects of electrochemotherapy on hepatic tissue, which requires further investigations. In contrast, IRE has been proved to be a potent and safe ablative method for inducing cell death up to the vessel wall without any perivascular sparing, as demonstrated in several animal studies as well as in human patients^8–10^. However, López-Alonso *et al*.^16^, who observed 6 pigs for 7 days after they were treated with IRE by using parallel-plate electrodes and a high-performance generator, detected the destruction of endothelial cells in some cases, followed by a repair processes beginning from the third day after IRE. Moreover, the authors identified the formation of intraluminal thrombi in some cases. Recently, a few studies that focus on vascular complications after IRE of hepatic malignancies in humans have been conducted. Narayanan *et al*.^10^ examined 158 vessels of 101 patients with 129 tumours (100 hepatic malignancies) after IRE. The follow-up controls were performed 1, 3, 6 and 12 months (mean: 10.3 months; range: 1–40 months) after treatment by using computed tomography or MRI. All the vessels were located 0–1.0 cm from the treatment zone. Venous alterations were detected in 7 vessels (4.4%), containing narrowing of the PV (n = 2) and the HV (n = 1) and thrombosis of the portal venous system (n = 4). The study of Distelmaier *et al*.^17^ did not show any venous complications in 43 hepatic tumours (29 patients) treated by IRE and immediately adjacent to major hepatic veins or portal vein branches. The follow-up controls were conducted by computed tomography or MRI 1, 2, 4, 6, 8 and 12 months after IRE and every 3 months thereafter (mean: 24 months). Dollinger *et al*.^11^ examined potential alterations in 191 venous structures of 43 patients suffering from hepatic tumours 1–3 days after ablation by IRE. All 191 vessels were located ≤ 1.0 cm from the ablation zone. 19 of the 191 vessels (9.9%) showed vascular changes, including partial or complete portal vein thrombosis (n = 5) or lumen narrowing (n = 14). In the follow-up control, thrombosis had resolved in 2 cases. Vessel narrowing had completely resolved in 8 cases, and partly resolved in 1 case.

As opposed to our study, in which we performed an intermediate-term follow-up (3 months) on all patients, Dollinger *et al*.^11^ only included patients with venous complications in the follow-up group. Consequently, a potential venous complication occurring for the first time at one of the later follow-up controls might not have been detected. Moreover, some of the named studies did not focus on one modality of imaging, varying between CT- and MR-imaging during follow-up, so that the evaluation of the analysing radiologist could have been influenced by the different modalities. The aim of the current study was to evaluate potential venous alterations after IRE at predefined points of time to illustrate a more accurate evolution during the whole period of follow-up.

**Figure 2:**
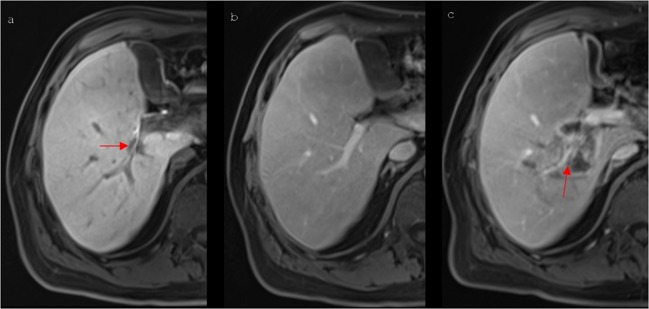
A 64-year old man with a centrally located HCC. (**a**) Pre-interventional Gd-EOB- DTPA-enhanced T1 vibe 3d fat suppressed magnetic resonance imaging in delayed phase shows a centrally located HCC (arrow) next to the portal vein encasing a bile duct. (**b**) The corresponding portal venous phase shows a freely perfused right branch of the portal vein. (**c**) 6 weeks after IRE contrast-enhanced T1 vibe 3d fat suppressed magnetic resonance imaging in portal venous phase shows a newly occurred partial thrombosis (arrow) of the right portal vein.

According to our results, the most frequently observed venous complication was thrombosis (47 of all 67 venous alterations; Table 4). 30 (63.8%) of all detected thrombosis were noted for the first time 6 weeks (n = 23) or 3 months (n = 7) after the intervention, indicating that thrombosis (Fig. 2) does not always represent an early complication but can occur delayed at a later stage. Although a significantly increased risk of delayed thrombosis in PVs could not been proven, the major proportion of thromboses occurred in the portal venous system (n = 35 in PVs; n = 12 in HVs). This outcome tends to coincide with the findings of Narayanan *et al*. and Dollinger *et al*., who described the occurrence of thrombosis solely in PVs^10,11^. A potential reason for PVs being affected more often by thrombosis after IRE are vessel-associated flow dynamics causing an increased fragility of this vessel type^10^. In the current study, post-ablative thrombosis could only be detected in vessels with a maximum diameter of 1.0 cm and a small vessel diameter represented a significant unfavourable prognosticator of delayed thrombosis (p = 0.012). The results suggest that small vessels next to an IRE ablation zone seem to be at an especially higher risk of developing post-interventional thrombosis. Consequently, it has to be contemplated whether an ongoing imaging follow-up or even prophylactic anticoagulation treatment is necessary in those patients. Although there were no veins with a distance of 0.1 cm or more from the ablation zone that were affected by thrombosis, no significant association between the development of post-ablative thrombosis and a closer local proximity was observed in the binary logistic regression model.

**Figure 3:**
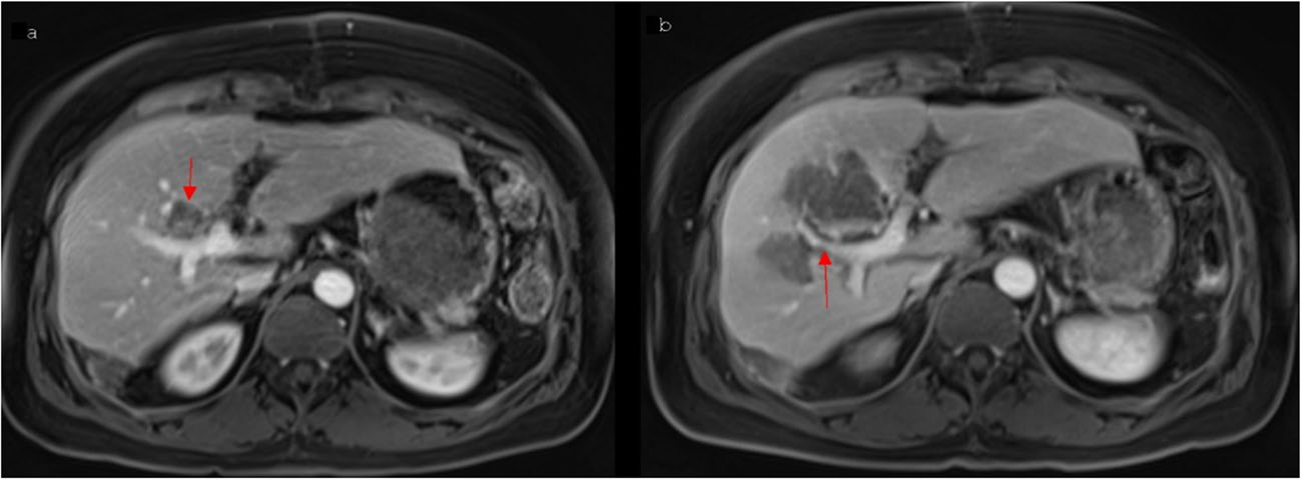
A 45-year old woman with a centrally located metastasis of colorectal cancer. (**a**) The Gd-EOB-DTPA-enhanced T1 vibe 3d fat suppressed magnetic resonance imaging in portal venous phase shows a centrally located metastasis (arrow) and a freely perfused right anterior branch of the portal vein. (**b**) 6 weeks after IRE imaging shows a newly occurred vessel narrowing of the portal vein branch (arrow).

20 of all 67 venous alterations were represented by vessel narrowing (Fig. 3), including high-grade stenosis in a few cases. 13 of them resolved during the first 3 months after IRE (Table 4), whereas 1 remained unchanged during the whole period of follow-up (for 1 case of vessel narrowing the follow-up controls 6 weeks and 3 months after IRE were missing). In 5 of 20 cases, vessel narrowing turned into thrombosis 6 weeks or 3 months after IRE. Pre-existing vessel narrowing was a significant risk factor for delayed thrombosis (p = 0.004), so it has to be considered whether prophylactic anticoagulation should be standard treatment for those patients. Only 1 patient showed a newly occurred, temporary, post-ablative vessel narrowing between 6 weeks and 3 months after the intervention. As a possible explanation for the temporary venous alterations, some authors assume the postinterventional oedematous, hyperaemic and inflammatory changes in ablated liver tissue^18–20^, whereas the single case of persistent vessel narrowing could be caused by post-ablative scarring^18,19^.

The present study has several limitations: The first is the retrospective nature of the study. The second limitation is the incomplete data acquisition of the 6 week- and 3 months follow-up control in 24 patients. Moreover, some of the patients had undergone an additional tumour treatment, i.e., chemotherapy or radiotherapy, which could have had a damaging effect on the observed vessels. Furthermore, the study group consisted of a heterogenous patient population in terms of sex and tumour type.

## Conclusions

The current study suggests that delayed venous thrombosis commonly occurs during the postinterventional period after IRE. Both pre-existing vessel narrowing and a small vessel diameter constitute significant risk factors, making imaging follow-up or even prophylactic anticoagulation necessary in those patients.

## Data Availability

The datasets generated during and/or analysed during the current study are available from the corresponding author on reasonable request.
